# Keratin 8 reduces colonic permeability and maintains gut microbiota homeostasis, protecting against colitis and colitis-associated tumorigenesis

**DOI:** 10.18632/oncotarget.18241

**Published:** 2017-05-27

**Authors:** Chao Liu, En-Dong Liu, Yun-Xiao Meng, Xiao-Ming Dong, Ya-Lan Bi, Huan-Wen Wu, Yan-Chao Jin, Ke Zhao, Jian-Jie Li, Miao Yu, Yi-Qun Zhan, Hui Chen, Chang-Hui Ge, Xiao-Ming Yang, Chang-Yan Li

**Affiliations:** ^1^ An Hui Medical University, Hefei, 230032, China; ^2^ State Key Laboratory of Proteomics, Beijing Proteome Research Center, Beijing Institute of Radiation Medicine, Beijing, 100850, China; ^3^ Department of Pathology, Peking Union Medical College Hospital, Chinese Academy of Medical Sciences and Peking Union Medical College, Tsinghua University, Beijing, 100730, China; ^4^ Tianjin University, School of Chemical Engineering and Technology, Department of Pharmaceutical Engineering, Tianjin, 300072, China; ^5^ Key Laboratory of Carcinogenesis and Translational Research, Ministry of Education, Department of Thoracic Oncology, Peking University Cancer Hospital Institute, Beijing, 100871, China

**Keywords:** CK8, colitis, colitis-associated colorectal cancer, gut microbiota, TLR4

## Abstract

Keratin 8 (CK8) is the major component of the intermediate filaments of simple or single-layered epithelia. Gene targeting mice model suggest that CK8 is involved in colonic active ion transport, colorectal hyperplasia and inflammation. In the present study, we found that CK8 is downregulated in the colon during DSS-induced colitis and AOM/DSS-induced colitis-associated colorectal cancer (CAC) development. In human patients with colon cancer, CK8 is downregulated. Using CK8 heterozygous knockout mice (CK8^+/−^), we found that CK8^+/−^ mice are highly susceptible to DSS-induced colitis and more prone to AOM/DSS-induced CAC than wild type (WT) mice. The colonic permeability is increased with DSS or AOM/DSS treatment, leading to alteration of gut microbiota in CK8^+/−^ mice with CAC. Metagenomic analysis of fecal microbiota suggests *Firmicutes* and *Proteobacteria* are increased in CK8^+/−^ mice with CAC, while *Bacteroidetes* and *Verrucomicrobia* are decreased. Antibiotic treatment decreases the incidence of colorectal cancer tumorigenesis and TLR4 inhibitor attenuates the susceptibility of CK8^+/−^ mice to DSS-induced colitis. These data suggest CK8 protects mice from colitis and colitis-associated colorectal cancer by modulating colonic permeability and gut microbiota composition homeostasis.

## INTRODUCTION

Chronic inflammation of the gut, such as that observed in inflammatory bowel disease (IBD), strongly predicts colitis-associated colorectal cancer (CAC) incidence and progression [1–3]. In their first 30 years with IBD, over a fifth of patients develop CAC, and half of these CAC cases are fatal [4]. Though the pathogenesis of IBD and CAC are not yet fully known, there is a general consensus that inflammatory conditions and the intestinal commensal microbiota composition are major contributors to the development of these diseases. Still, there is a great need for research into the regulation of these factors.

In the majority of eukaryotic cell types, intermediate filaments (IFs) are key constituents of the nuclear envelope and cytoskeleton [5]. The keratin subfamily, expressed specifically by epithelial cells, comprises more than 20 proteins (keratins 1-20). Type I proteins (keratins 9-20) pair with type II proteins (keratins 1-8), forming obligate noncovalent heteropolymers [6]. In the exocrine pancreas, liver, gastrointestinal tract and mammary gland, the simple or single-layered epithelia (where carcinomas frequently arise) contain IFs predominantly composed of keratins 8 and 18 (CK8/18) [7].

Animal studies have indicated that CK8 is involved in the development of IBD. One report [8] demonstrated that homozygous CK8^−/−^ FVB/N mice developed colitis, colonic hyperplasia and rectal prolapse. Hyperplasia was evident in histological analyses, while the crypt cells and mucin-producing goblet cells appeared normal; thus, colorectal hyperplasia seemed to be responsible for the bowel inflammation in CK8^−/−^ mice [8]. Chronic T helper type 2 colitis (Th2 colitis) was also reported to occur spontaneously in CK8^−/−^ mice [9]. Immunohistochemical and flow cytometric analyses revealed that the infiltration of T-cell receptor beta (TCRβ)-positive CD4^+^ T cells into the lamina propria of the colon, as well as the production of Th2 cytokines (IL-4, IL-5 and IL-13), was elevated in CK8^−/−^ mice. However, antibiotics such as imipenem and vancomycin inhibited the inflammation of the CK8^−/−^ colon, indicating that bacteria in the lumen may induce colitis when there is a primary epithelial defect in mice [9]. In addition, the active transport of ions (sodium and chloride) in the colons of CK8-null (CK8^−/−^ and CK8^+/−^) mice differed from that of CK8^+/+^ mice [10]. The short circuit current (I_SC_) was also significantly reduced in CK8-null mice; however, their colon tissues exhibited normal paracellular transport and conductance [10]. This indicated that keratins may regulate ion transport proteins and the transport of electrolytes in colonocytes [10].

A subgroup of IBD patients was recently reported to have missense mutations in CK8 [11]. In a proteomic study of patients with colorectal cancer (CRC), polyps or no pathology, certain isoforms of CK8 were expressed at higher levels in morphologically normal mucosa from polyp and cancer patients than in those from healthy subjects [12]. CK8 expression in CRC was also reported to correlate directly with survival, potentially because of its relationship to the epithelial-mesenchymal transition [13]. All these data suggest that CK8 is involved in barrier protection, colonic active ion transport, colorectal hyperplasia and inflammation. However, the involvement of CK8 in inflammation-associated CRC remains poorly understood.

Embryonic lethality has been reported to occur in about 50% of homozygous CK8 knockout mice. Our previous study demonstrated that CK8 was completely abolished in CK8^−/−^ mice, while CK8^+/−^ mice had about half the CK8 level of CK8^+/+^ mice in all examined tissues [14]. Furthermore, CK8^+/−^ mice were phenotypically similar to wild-type (WT) controls, with only slight crypt proliferation and partial ion transport dysfunction [10]. Only 3% of heterozygous mice exhibited anorectal prolapse. These data suggest that CK8^+/−^ mice are a usable model mimicking CK8 knockout mice.

In the present study, we have examined CK8 expression in experimental colitis and CAC models and in clinical patients with colon cancer. Using CK8^+/−^ mice, we have investigated the effects of CK8 on the gut microbiota composition and intestinal permeability during dextran sodium sulfate (DSS)-induced colitis and azoxymethane (AOM)/DSS-induced CAC. Finally, we have assessed the influence of antibiotics and a toll-like receptor 4 (TLR4) inhibitor on CRC tumorigenesis and DSS-induced colitis, respectively, in CK8^+/−^ mice.

## RESULTS

### CK8 is downregulated in colitis and CAC

Colorectal cancer risk is elevated in ulcerative colitis patients [15, 16]. CK8 is the major component of the IFs in the gut epithelium, and is downregulated in CRC [7]. The absence of CK8 in mice stimulates the development of colonic hyperplasia and colitis[8]. These data suggest that CK8 might be important for preventing CAC.

**Figure 1:**
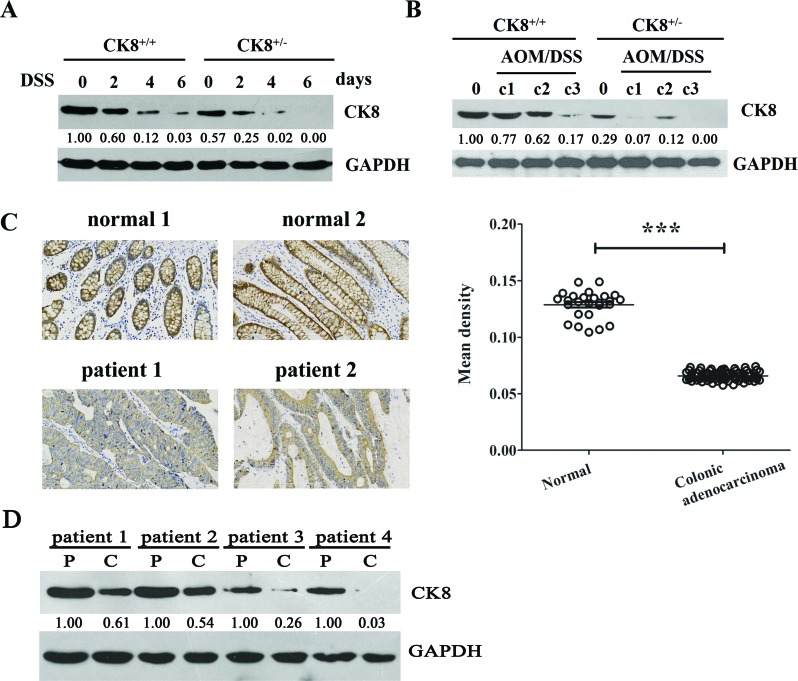
CK8 is down regulated in colitis and colitis-associated colorectal cancer (**A**) CK8^+/+^ mice and CK8^+/–^ mice were fed with 5% DSS-containing drinking water for 5 consecutive days and followed by regular water for 1 day. The colonic expression of CK8 were investigated using Western blotting analysis. The blots bands were scanned for densitometry analysis with the value obtained from CK8^+/+^ mice without DSS treatment set as 1. (**B**) Colitis- associated colorectal cancer was induced in CK8^+/+^ mice and CK8^+/−^ mice with AOM/DSS treatment as Material and Methods described. The colonic expression of CK8 were investigated using Western blotting analysis. The blots bands were scanned for densitometry analysis with the value obtained from CK8^+/+^ mice without AOM/DSS treatment set as 1. (**C**) The expression level of CK8 in human specimens of normal (*n* = 26) and neoplastic colon (*n* = 76) using immunohistochemical analysis with a specific antibody to the CK8 protein. The Mean density of CK8 positive cells in each sample was quantified by Image Pro-plus 6.0. ^***^P < 0.001.(**D**) The colonic expression of CK8 in four colon cancer patients were investigated using Western blotting analysis(P: para-cancer tissues, C:cancer tissues). The blots bands were scanned for densitometry analysis with the value obtained from colon cancer patients’ para-cancer tissues set as 1.

We first detected the expression of colonic CK8 during DSS-induced acute colitis. Age- and sex-matched CK8^+/+^ and CK8^+/−^ mice were treated with 5% DSS for 5 days, and colonic CK8 expression was examined. As shown in Figure 1A, colonic CK8 expression was significantly reduced in the CK8^+/+^ mouse following colonic injury with DSS, and was almost undetectable in CK8^+/−^ mice.

We then treated mice with AOM/DSS, a well-established method of inducing CAC [9]. Consistent with previous reports [17, 18], when WT mice were intraperitoneally injected with a single dose of AOM (a carcinogen) and given three cycles of 2% DSS in their drinking water, they developed multiple middle- to distal-colon tumors (data not shown). We next assessed the colonic expression of CK8 during CAC. As shown in Figure 1B, CK8 expression was significantly reduced in both CK8^+/+^ and CK8^+/−^ mice after AOM/DSS treatment; however, by the end of the third cycle, the expression of CK8 was almost undetectable in CK8^+/−^ mice.

Given the above observations, we predicted that CK8 expression might decrease during the development of colonic inflammation and colon cancers. Thus, we performed immunohistochemistry to determine the levels of CK8 in normal and neoplastic human colon specimens (*n* = 102) with a specific antibody against CK8. The specificity of the CK8 antibody is shown in Supplementary Figure 1. The results demonstrated that the surface epithelial cells and crypt cells of the normal colonic mucosa expressed high levels of CK8. On the other hand, in colonic adenocarcinoma specimens, CK8 expression was dramatically reduced (Figure 1C). We also assessed CK8 protein levels in specimens from four colon cancer patients. As expected, CK8 levels were significantly lower in cancer tissues than in para-cancer tissues (Figure 1D).

Taken together, these results indicate that CK8 is downregulated in colorectal tumors and may be important for preventing CAC tumorigenesis.

### Knockdown of CK8 promotes susceptibility to AOM/DSS-induced CAC

**Figure 2:**
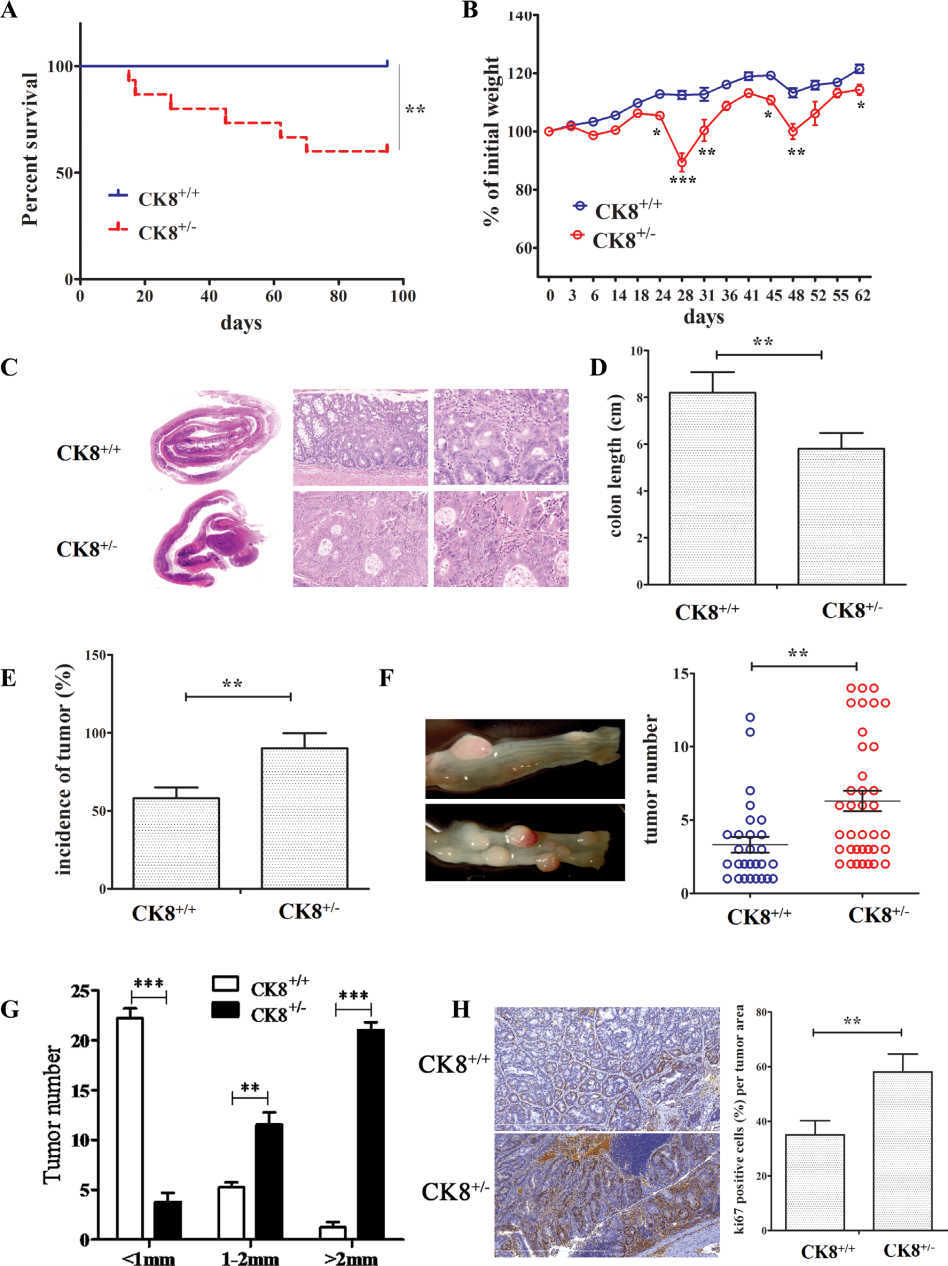
Knockdown of CK8 promotes susceptibility to AOM/DSS-induced colitis-associated colorectal carcinoma (**A**) CK8^+/+^ mice and CK8^+/−^ mice were treated with AOM/DSS as Material and Methods described. Their survival was monitored until day 96 after treatment with AOM. Survival Differences were evaluated with the Mantel-Cox test. ^**^P < 0.01. (**B**) The mean changes in body weight of the CK8^+/+^ and CK8^+/−^ mice were measured at the indicated time until day 62. At the end of 2nd cycle DSS on day 45 full length of the colon was prepared in a Swiss roll method and subject to H&E staining (**C**). The colon length was measured (**D**). At the time of harvest after 3rd DSS cycle, incidence of macroscopic polyps was analyzed (**E**). (**F**) Tumors within the colon were counted with the assistance of stereomicroscopy. (**G**) Measurement of largest dimension of tumor (mm) was performed using calipers. (**H**) Ki-67 immunohistochemistry staining (left panel) and percentage of Ki-67 positive cells. Data are shown as the mean ± s.d and are representative of three independent experiments. ^*^P < 0.05, ^**^P < 0.01, ^***^P < 0.001.

We further examined the vulnerability of CK8^+/+^ and CK8^+/−^ mice to AOM/DSS-induced CAC tumorigenesis, using a previously reported method [19]. During AOM and DSS treatment, CK8^+/−^ mice exhibited greater mortality than WT mice (Figure 2A), and by day 95, about 40% of the CK8^+/−^ mice had died. CK8^+/−^ mice also exhibited accelerated weight loss during the DSS treatment cycles (Figure 2B).

While CK8^+/+^ and CK8^+/−^ mice treated with AOM and DSS each developed colonic tumors, the tumor incidence was significantly greater in CK8^+/−^ mice than in WT mice after the second cycle of DSS treatment (Figure 2C), and the colon length was shortened in the CK8^+/−^ group (Figure 2D). At the time of harvest after the third DSS cycle, about 90% of the CK8^+/−^ mice had developed tumors, whereas only 58% of the WT mice exhibited macroscopically visible adenoma lesions (Figure 2E). We counted and measured macroscopic tumors over the entire length of the colon and rectum. As displayed in Figure 2F and 2G, CK8^+/−^ mice developed significantly more tumor lesions than WT mice. The colon and rectal tumors of the CK8^+/−^ mice were threefold greater in volume than those of the WT mice. While tumors were mostly found in the colorectal and distal portions of the WT colon, tumors were frequently observed along the whole length of the CK8^+/−^ colon.

**Figure 3:**
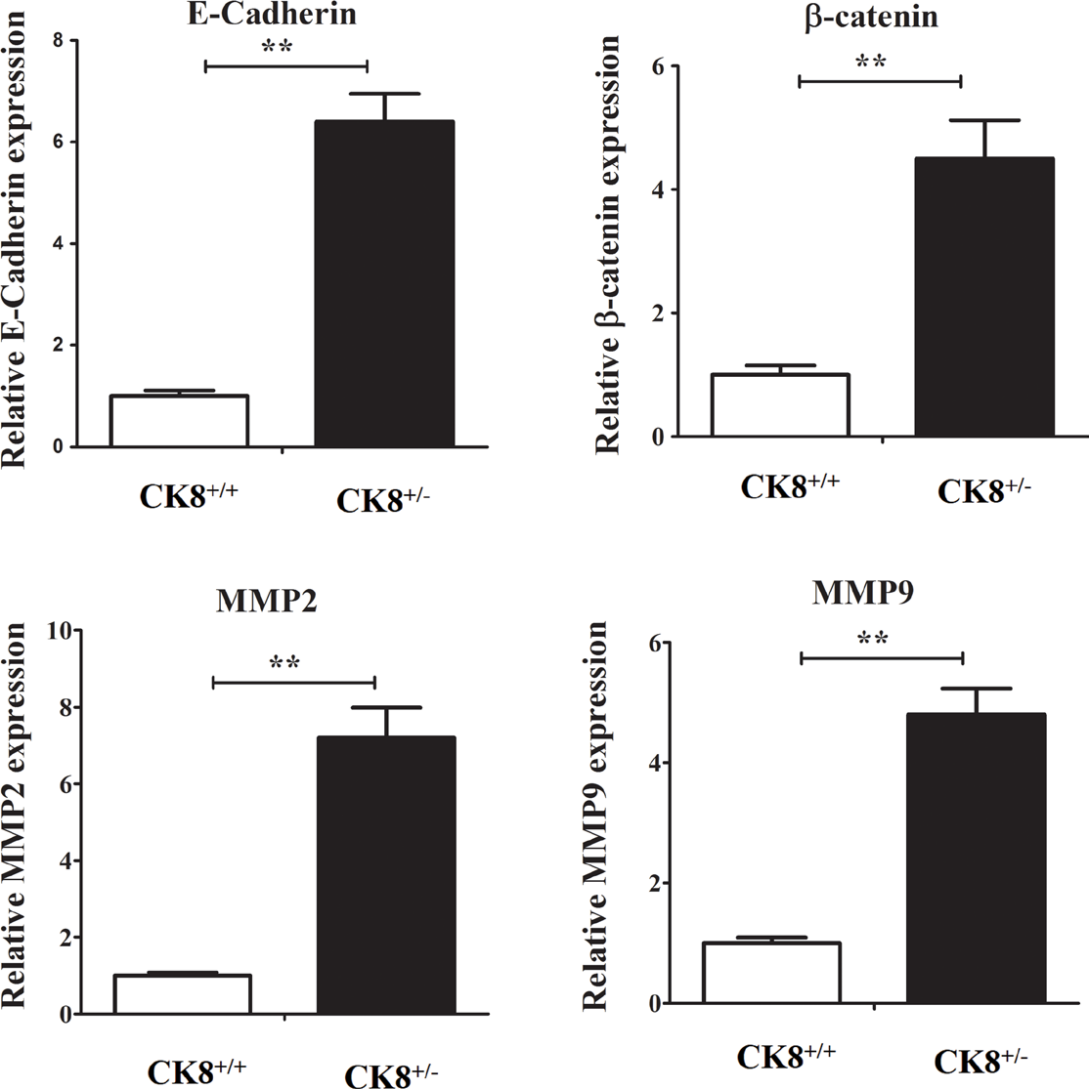
Cancer-related genes mRNA expression in colons of CK8^+/+^ mice and CK8^+/−^ mice with colorectal cancer CK8^+/+^ mice and CK8^+/−^ mice were treated with AOM/DSS as the above described and then RNA was extracted for real-time PCR analysis. Data are shown as the mean ± s.d and are representative of five individuals. ^**^P < 0.01.

We also counted Ki-67-stained cells, and found a larger number of positive cells in CK8^+/−^ tumors than in WT tumors (Figure 2H), suggesting that the downregulation of CK8 enhances the proliferation of tumor cells. However, the extent of apoptosis did not differ in tumorous colons from CK8^+/−^ and WT mice (Supplementary Figure 2). The mRNA levels of cancer-related genes including *β-catenin*, *E-cadherin*, *Mmp2 and Mmp9* were higher in CK8^+/−^ mice than in WT mice (Figure 3).

These observations suggest that the downregulation of CK8 increases the vulnerability of mice to CAC tumorigenesis.

### CK8 inhibits DSS-induced inflammatory responses

**Figure 4:**
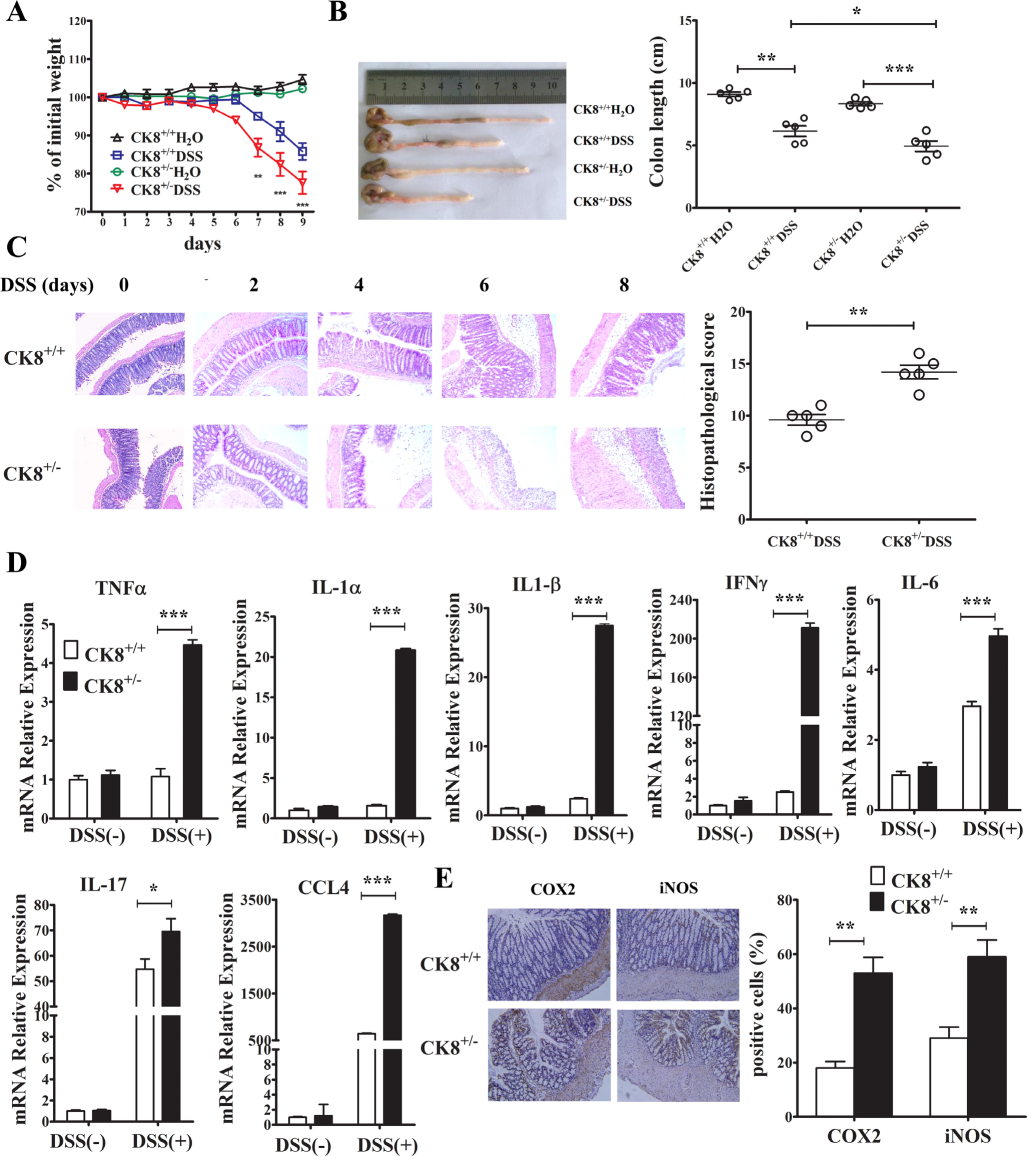
CK8 dampens inflammatory responses after DSS induction (**A**) The mean changes in body weight of the CK8^+/+^ and CK8^+/−^ mice (*n* = 10 for each genotype) after being fed with 5% DSS were measured every day until day 9. Colon length were measured (**B**). Histological changes in the colon tissue were examined by H&E staining (**C**). Inflammation score of colon tissue was performed as described in materials and methods. (**D**) The levels of the indicated genes in colons from CK8^+/+^ or CK8^+/−^ mice were quantified by real-time PCR analysis (*n* = 3 for each group). (**E**) The levels of COX2 and iNOS in colon tissue (*n* = 5 for each group) mice were measured using immunohistochemistry staining. Data are shown as the mean ± s.d. ^*^P < 0.05, ^**^P < 0.01, ^***^P < 0.001.

Previous studies have suggested that inflammatory conditions are key contributors to CAC tumorigenesis. We therefore hypothesized that CK8^+/−^ mice may have been more susceptible to CAC induced by AOM/DSS because of an enhanced inflammatory response to DSS treatment. First, we investigated the influence of CK8 downregulation on the severity of colitis induced by DSS. We treated CK8^+/+^ and CK8^+/−^ mice for 5 days with 5% DSS, then for 3 days with normal drinking water. While DSS induced weight loss in both CK8^+/+^ and CK8^+/−^ mice, a greater extent of weight loss was apparent in CK8^+/−^ mice by day 7 (Figure 4A). By the ninth day, around 90% of the CK8^+/−^ mice were hunched over, had lost roughly 30% of their body weight, and needed to be euthanized. Although we observed malformed stools and colonic edemas during necropsies of both CK8^+/+^ and CK8^+/−^ mice, the CK8^+/−^ mice had shorter colons, indicating that their colonic injuries were more severe (Figure 4B). When we examined the histology of Swiss-rolled colons stained with H&E, CK8^+/−^ colons mainly exhibited distal injuries including epithelial ulcers, submucosal edemas and inflammatory infiltrates, indicating that more severe injuries had occurred in the CK8^+/−^ mice (Figure 4C).

We further investigated the expression of various inflammatory cytokines and chemokines in WT and CK8^+/−^ mice. The levels of *Tnf*α*, Ifnγ, Il-1*α*, Il-1*β*, Il-17, Ccl4* and *Il-6* were elevated in colon tissues from CK8^+/−^ mice (Figure 4D). Additionally, the levels of typical inflammation markers (nitric oxide synthase (NOS) and cyclooxygenase 2 (COX2)) were greater in CK8^+/−^ colons than in WT colons after DSS treatment (Figure 4E). Only the colon (especially the distal portion) exhibited this inflammation, while little inflammation was observed in the small intestine (data not shown). These data are consistent with previous reports and suggest that the downregulation of CK8 stimulates inflammation in DSS-stimulated acute colitis.

**Figure 5:**
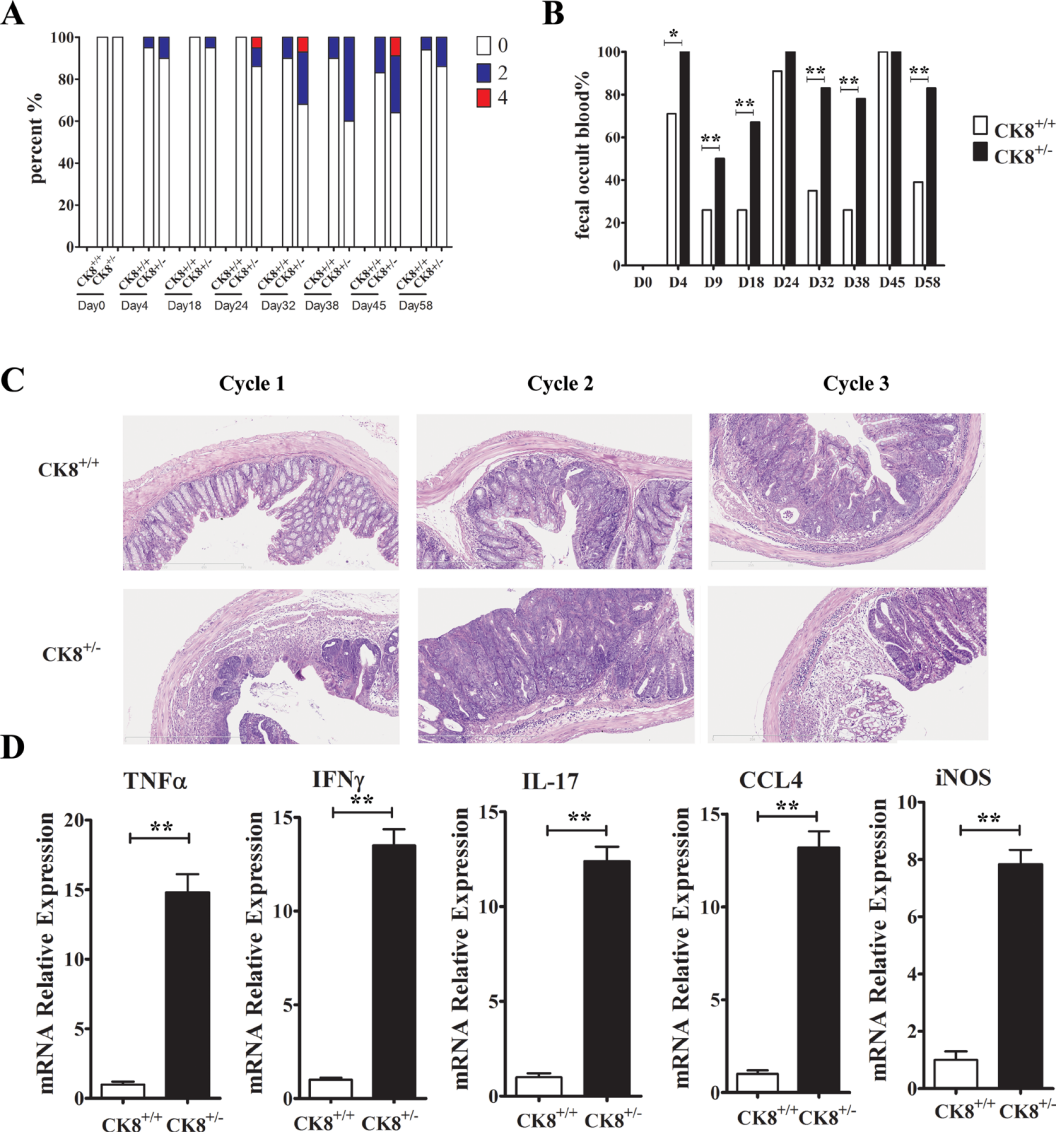
Increased inflammatory response in AOM/DSS-treated CK8^+/−^ mice compared to CK8^+/+^ mice (**A**) CK8^+/+^ mice and CK8^+/−^ mice (*n* = 12 for each group) were treated with AOM/DSS as the above described. The diarrhea score (A), the percent of fecal occult blood positive mice (**B**) were analyzed. Histological changes in the colon tissue were examined at the end of each DSS cycle (**C**). (**D**) The mRNA expression of inflammatory cytokines indicated were quantified by real-time PCR analysis (*n* = 3 for each group). Data are shown as the mean ± s.d. ^*^P < 0.05, ^**^P < 0.01.

We next evaluated the inflammatory responses of AOM/DSS-treated mice. More severe colitis was detected in CK8^+/−^ mice than in WT mice, as evidenced by their greater weight loss (Figure 2B), more severe diarrhea (Figure 5A) and fecal occult blood (Figure 5B) after DSS treatment. Following long-term DSS treatment, CK8^+/−^ mice had significantly shorter colons than WT mice (Figure 2D). We also evaluated the histopathology of the colon in the early phase of tumor induction (15 days following the injection of AOM). As shown in Figure 5C, the extent of inflammation, mucosal edema, tissue damage, and hyperplasia was significantly greater in CK8^+/−^ colons than in WT colons. Then, we evaluated the mRNA levels of various cytokines and inflammatory factors in colon tissues from CK8^+/−^ and WT mice with tumors. As shown in Figure 5D, proinflammatory cytokines such as *Tnfa*, *iNos*, *Ifng* and *Il-17* and the chemokine *Ccl4* were significantly upregulated in CK8^+/−^ colons compared to WT colons.

All these data suggest that CK8 is important for dampening inflammatory responses during AOM/DSS-induced CAC tumorigenesis.

### CK8^+/−^ mice exhibit increased colonic permeability during DSS-induced colitis and AOM/DSS-induced colorectal carcinogenesis

TNFα has been shown to be critical for the initiation and progression of colitis and CAC carcinogenesis, and blocking TNFα in mice reduced colorectal carcinogenesis with colitis following AOM and DSS treatment [20]. Since TNFα was upregulated in CK8^+/−^ mice after DSS treatment, it was of interest to determine if blocking TNFα would reduce the colorectal carcinogenesis in CK8^+/−^ mice. We administered a specific TNFα antagonist (etanercept) to CK8^+/−^ mice that had been treated with DSS; however, etanercept did not reduce the incidence of tumorigenesis (data not shown), suggesting that TNFα is not essential for AOM/DSS-induced colorectal carcinogenesis in CK8^+/−^ mice.

**Figure 6:**
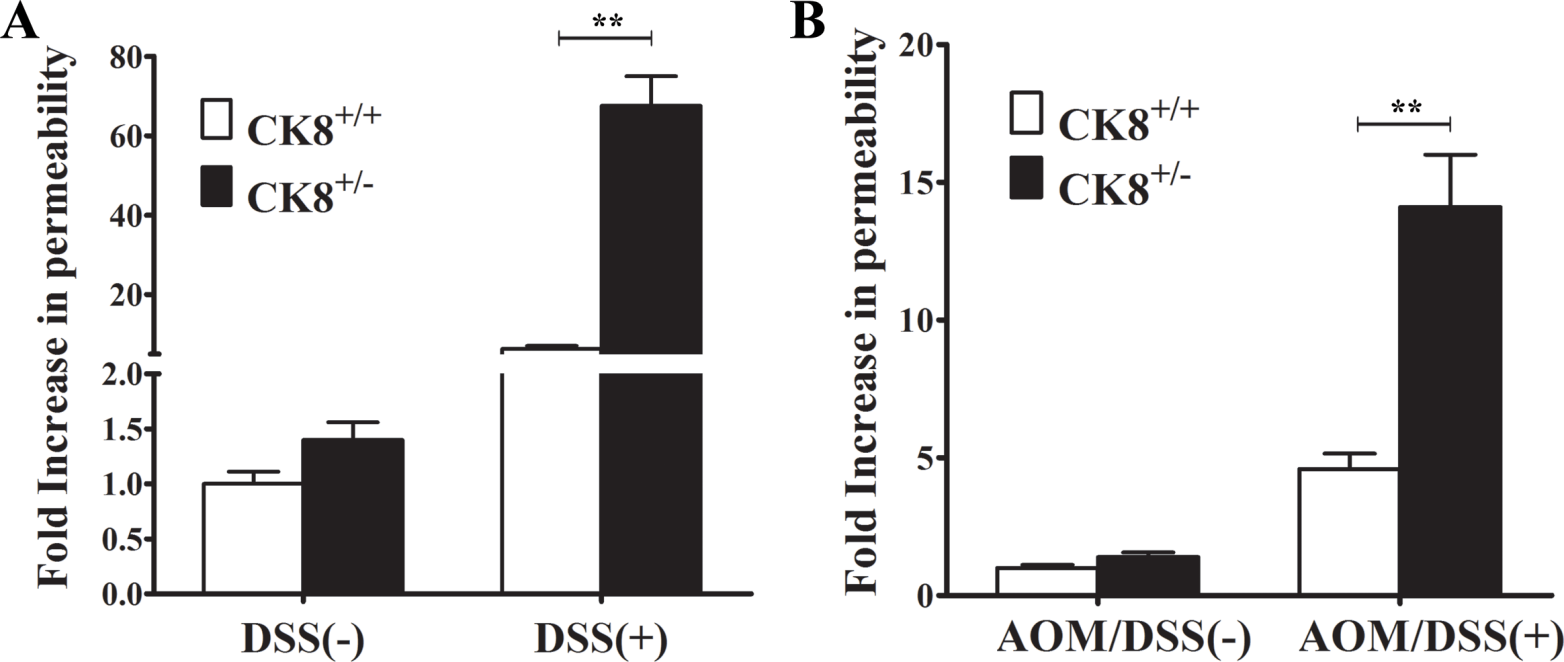
CK8^+/−^ mice exhibit increased colonic permeability during DSS-induced colitis and AOM/DSS-induced colorectal carcinogenesis (**A**) CK8^+/+^ mice and CK8^+/−^ mice were treated with 5% DSS as above described and on day 7 FITC-dextran permeability assay was performed (*n* = 4 for each group). (**B**) CK8^+/+^ mice and CK8^+/−^ mice were treated with AOM/DSS as above described and on day 60 at the end of 3rd DSS cycle FITC-dextran permeability assay was performed (*n* = 4 for each group). Data are shown as the mean ± s.d. ^**^P < 0.01.

A previous study suggested that CK8, together with IL-6, protects the epithelial barrier from direct luminal damage (such as that from DSS) and the accompanying inflammatory response [15]. We therefore used the FITC-labelled dextran method to investigate the colonic permeability of CK8^+/−^ mice treated with DSS or AOM/DSS. Since the gut does not actively absorb FITC-dextran, intestinal permeability can be assessed directly *in vivo* based on the amount of fluorescence in the serum. FITC-dextran was administered to CK8^+/−^ mice and WT mice on day 7 after 5% DSS treatment and on day 60 at the end of the third DSS cycle. As shown in Figure 6A, under conditions with normal drinking water, colonic permeability did not differ significantly between WT and CK8^+/−^ mice, whereas with DSS or AOM/DSS treatment, CK8^+/−^ mice demonstrated greater colonic permeability (higher levels of fluorescence in the serum) than WT mice. In the presence of 5% DSS for 7 days, the colonic permeability was about 10-fold greater in CK8^+/−^ mice than in WT mice. In the CAC model with AOM/DSS treatment, CK8^+/−^ mice displayed 3-fold greater permeability than WT mice (Figure 6B). These results suggest that the greater colonic inflammation and tumor development in CK8^+/−^ mice is associated with greater colonic permeability.

### Fecal microbiota of CK8^+/−^ mice differ from those of WT mice after AOM/DSS treatment

We next examined whether CK8^+/−^ mice predisposed to developing CAC had different colonic microbiota than WT mice. No obvious difference in fecal microbiota composition was observed between CK8^+/−^ mice and WT mice. Then, we treated age-matched (8 weeks) and sex-matched CK8^+/−^ and littermate WT mice with AOM/DSS in the same manner discussed above, and harvested fecal samples at the end of the third DSS cycle. The 16S ribosomal RNA gene in the fecal samples was sequenced. All the analyzed sequences belonged to Kingdom Bacteria and were assigned to 18 phyla, including *Bacteroidetes*, *Firmicutes*, *Verrucomicrobia*, and *Proteobacteria*, encompassing the majority of sequences (> 97%).

**Figure 7:**
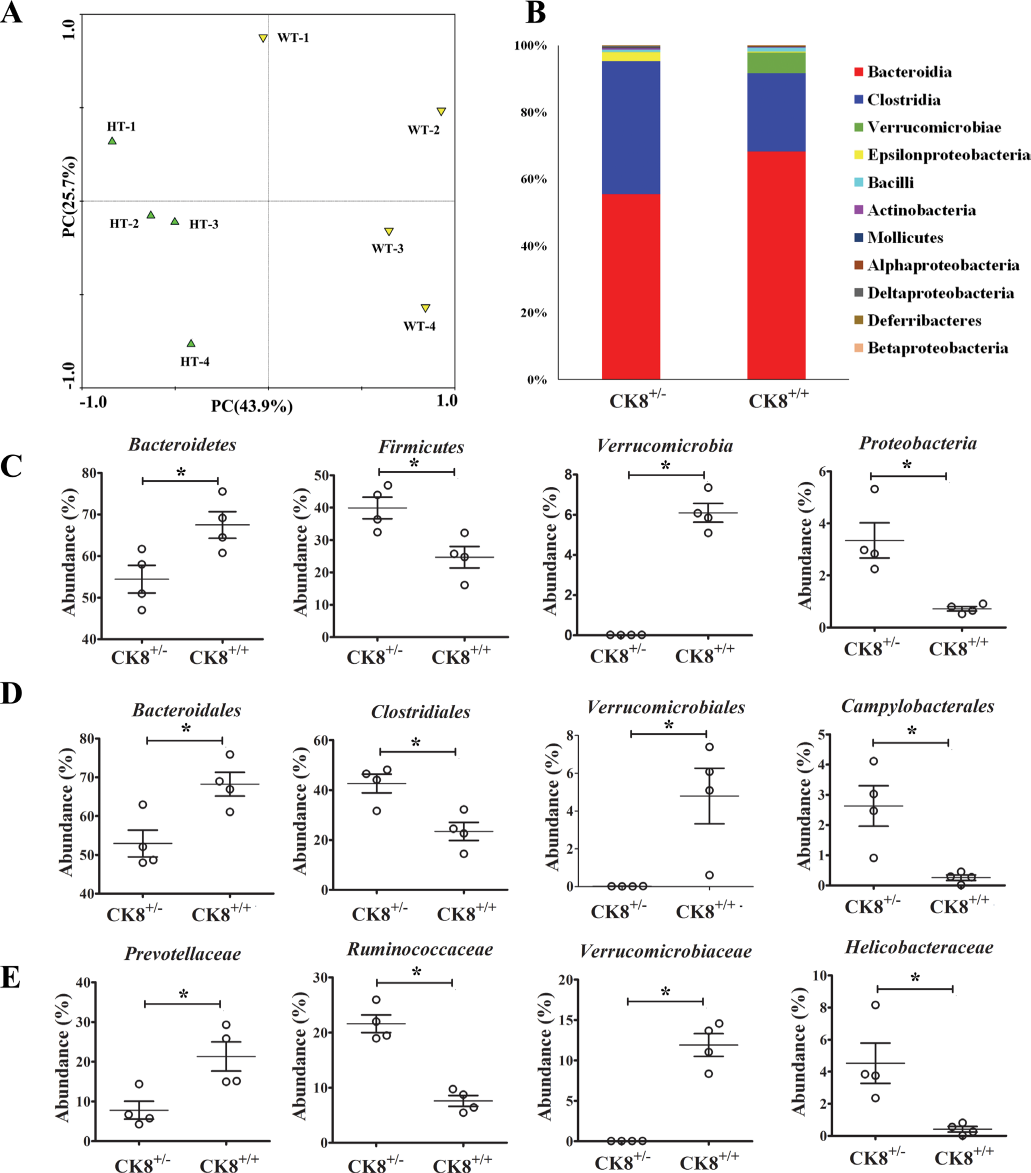
Fecal microbiota of CK8^+/−^ mice is distinguished from that of WT mice after AOM/DSS treatment The age-matched (8 weeks) and sex-matched CK8^+/−^ and littermate WT mice were treated with AOM/DSS as described above, then at the end of the 3rd DSS cycle, fecal samples were harvested. The 16S ribosomal RNA gene in the fecal samples was sequenced. (**A**) Principal coordinates analysis (PCA) scores plot based on the relative abundance of OTUs (97% similarity level). (**B**) Histogram represents the relative abundance of bacterial class in each group (*n* = 4 for each group). The abundance of major bacteria at the phylum (**C**), order (**D**), and family (**E**) was analyzed. Each dot represents an individual microbiota sample from the mice. *Horizontal bars* indicate mean values. ^*^P < 0.05, Mann-Whitney *U* test. *n* = 4 per group.

From a principal coordinates analysis plot (Figure 7A) based on the predominant genera, we determined that CK8^+/−^ mice and WT mice each formed a strong single cluster. The fecal microbiota from CK8^+/−^ and WT mice were easily distinguished within the primary 3-axis. The taxa that differed most between the two communities are shown in Figure 7B. *Clostridia* and *Epsilonproteobacteria* were enriched in CRC CK8^+/−^ mice, whereas *Bacteroidia* and *Verrucomicrobia* were enriched in CRC WT mice; these were all important class types for segregating the fecal microbiota of WT and CK8^+/−^ mice. These data suggest that the fecal microbiota communities are indeed different in AOM/DSS-treated CK8^+/−^ and WT mice.

We further compared the gut microbiota between CRC WT mice and CRC CK8^+/−^ mice at different levels. As shown in Figure 7C, at the phylum level, *Bacteroidetes* and *Verrucomicrobia* were more abundant in the gut microbiota of CRC WT mice than in those of CRC CK8^+/−^ mice. On the other hand, the abundance of *Firmicutes* and *Proteobacteria* was significantly lower in CRC WT mice. At the family level, *Bacteroidaceae*, *Verrucomicrobiaceae* and *Prevotellaceae* were relatively more abundant in CRC WT mice, while *Lachnospiraceae*, *Ruminococcaceae* and *Helicobacteraceae* were more abundant in CRC CK8^+/−^ mice (Figure 7D). At the genus level, genera *Alloprevotella*, *Oscillibacter*, and *Helicobacter* were all enriched in the feces of CRC CK8^+/−^ mice compared with CRC WT mice (Figure 7E), whereas *Bacteroides* and *Akkermansia* were enriched in CRC WT mice. Other differences between the WT and CK8^+/−^ mice are shown in Supplementary Table 2.

### Intestinal depletion of bacteria reduces tumor formation in CK8^+/−^ mice

**Figure 8:**
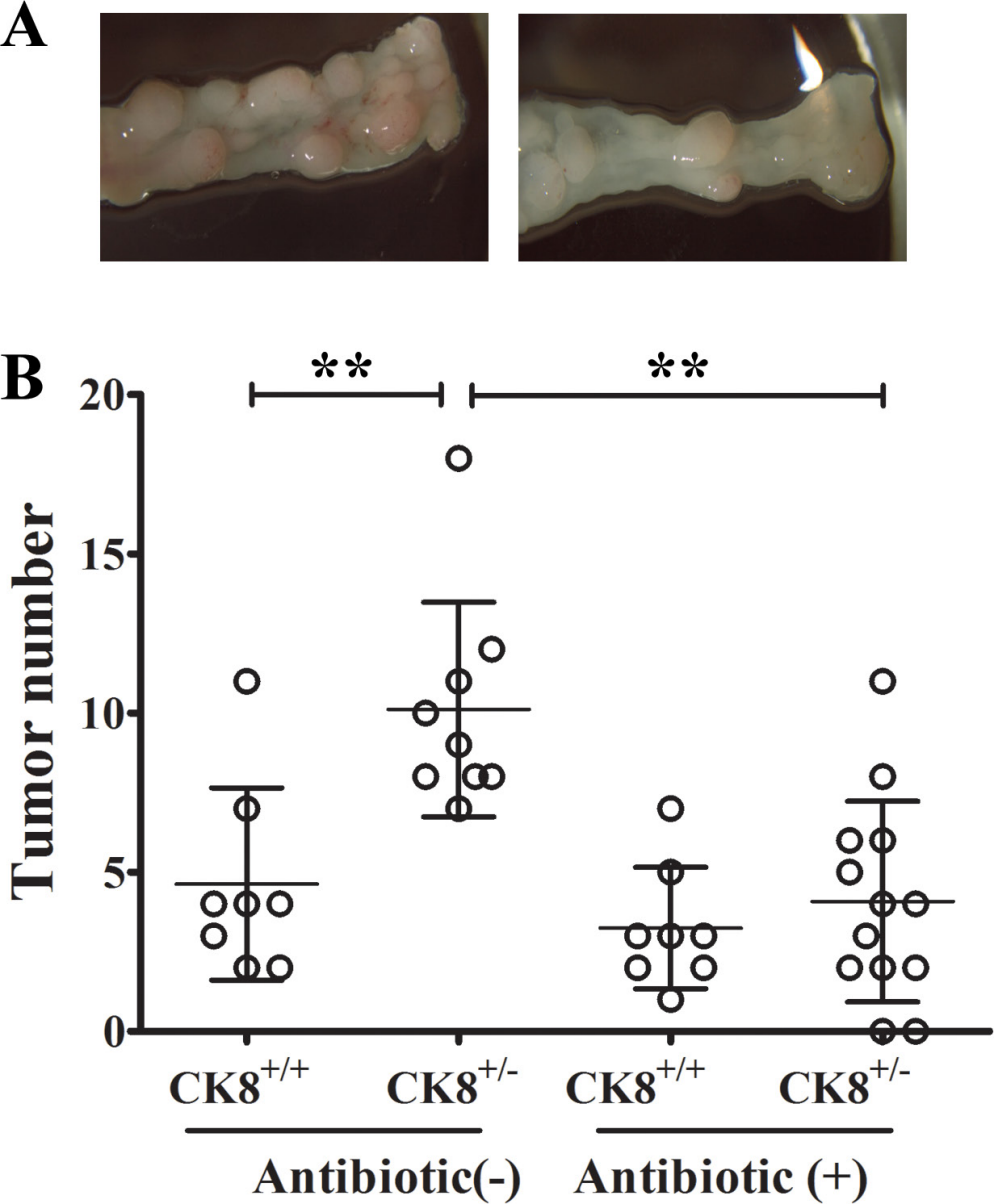
Intestinal depletion of bacteria reduces tumor formation in CK8^+/−^ mice CK8^+/−^ and CK8^+/+^ mice were treated with an antibiotic cocktail for one month and then induced to colorectal cancer with AOM/DSS. Tumors within the colon were counted. CK8^+/−^ mice treated with antibiotic group, *n* = 13; other groups, *n* = 8. Data are shown as the mean ± s.d. ^**^P < 0.01.

The pathogenesis of human IBD [21, 22] and colon tumorigenesis [23] have recently been reported to be profoundly influenced by altered gut microbiota. Since CK8^+/−^ mice exhibited greater colonic permeability and different gut microbiota than WT mice following AOM/DSS treatment, we evaluated whether the greater sensitivity of the CK8^+/−^ mice to CAC depended on commensal bacteria. CK8^+/−^ mice were treated with an antibiotic cocktail for one month, which remarkably depleted their anaerobic intestinal bacteria (the majority of the gut microbiota; data not shown). Then, AOM/DSS was used to stimulate tumorigenesis, as previously described. Consistent with previous reports, the administration of an antibiotic cocktail increased the mortality following DSS treatment, confirming the importance of the gut microbiota for intestinal homeostasis [24, 25]. As seen in Figure 8A and 8B, the depletion of intestinal bacteria by antibiotic treatment of CK8^+/−^ mice dramatically suppressed colon tumor formation.

These results indicate that the gut microbiota might have contributed to the increased colitis and colitis-associated tumorigenesis in CK8^+/−^ mice.

### TLR4 inhibitor treatment reduces the vulnerability of CK8^+/−^ mice to colitis induced by DSS

**Figure 9:**
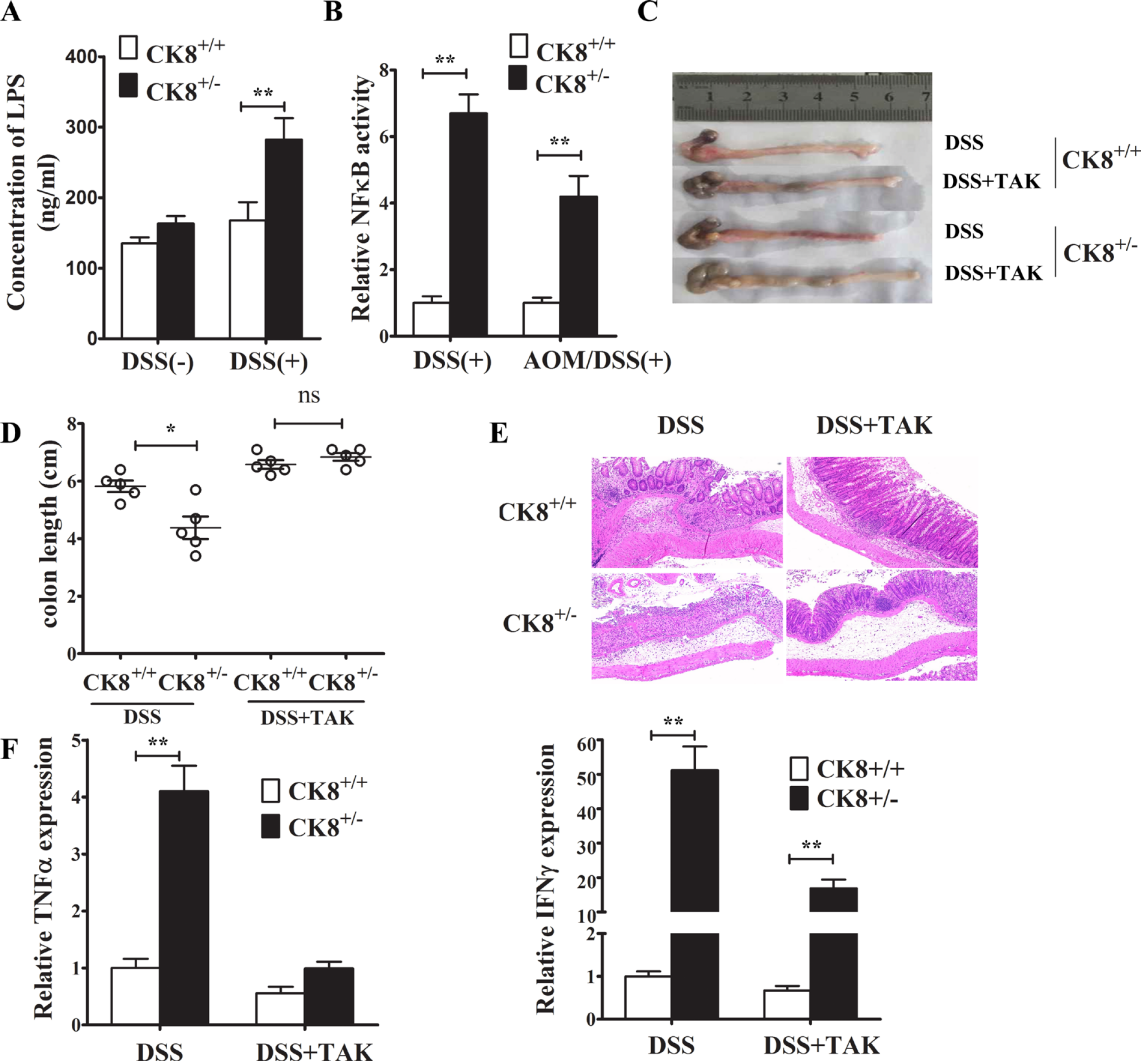
TLR4 inhibitor treatment reduced the susceptibility of CK8^+/−^ mice to DSS-induced colitis CK8^+/−^ and CK8^+/+^ mice were treated with DSS as above described. The serum lipopolysaccharide (LPS) level was investigated (**A**). (**B**) NF-κB activity in colonic tissue from DSS or AOM/DSS treated CK8^+/−^ and CK8^+/+^ mice were measured.(**C**) TAK-242 were administered intravenously at the dose of 1mg/kg to CK8^+/−^ and CK8^+/+^ mice. Then the mice were fed 5% DSS-solution in drinking water for 5 days, followed by regular drinking water for 3 days. The severity of colitis was evaluated with colon length (**D**), histological injury (**E**), and expression levels of inflammatory cytokines (**F**). Data are shown as the mean ± s.d. ^*^P < 0.05, ^**^P < 0.01. *n* = 5 for each group.

As described above, the phylum *Proteobacteria* was significantly upregulated in CRC CK8^+/−^ mice. Within *Proteobacteria*, the *Epsilonproteobacteria* class, *Campylobacterales* order and *Helicobacteraceae* family were all significantly more abundant in CRC CK8^+/−^ mice than in CRC WT mice. Previous studies have suggested that *Proteobacteria* species are elevated in colitis [26] and CAC [27]. Infection with different *Helicobacter spp.* led to CAC development, suggesting that *Helicobacter spp.* in particular promote CRC tumorigenesis [28]. Given that *Helicobacteraceae* are classified as gram-negative bacteria, we investigated the lipopolysaccharide (LPS) levels from these bacteria in the serum of DSS-induced CK8^+/−^ mice and WT mice. As shown in Figure 9A, during DSS-induced colitis, the LPS levels were significantly higher in CK8^+/−^ mice than in WT mice.

Our previous study suggested that CK8 suppressed TLR/Nuclear Factor Kappa B (NF-kB) signaling by preventing the polyubiquitination of TNF Receptor Associated Factor 6 (TRAF6). Thus, we investigated the transcriptional activity of NF-kB in colonic tissue from mouse models of DSS-induced acute colitis and AOM/DSS-induced CRC. As expected, colonic NF-κB activity in DSS-induced colitis and CRC was significantly higher in CK8^+/−^ mice than in WT mice (Figure 9B), suggesting that the downregulation of CK8 increases the activation of NF-κB in association with more severe inflammation.

We further assessed whether TLR signaling enhanced the vulnerability of CK8^+/−^ mice to DSS-induced colitis. CK8^+/−^ mice and WT mice were treated with TAK-242 (a small-molecule inhibitor that specifically binds to the intracellular domain of TLR4 and thus prevents LPS from stimulating the generation of inflammatory mediators), given a 5% DSS solution in their drinking water for 5 days, and then given normal drinking water for 3 days. The severity of colitis was evaluated based on the colon length, histological injury score, and inflammatory cytokine expression. As shown in Figure 9C and 9D, the shorter colon length in the CK8^+/−^ strain after DSS treatment was reversed by TAK-242 treatment. When we histologically analyzed colons stained with H&E, we found further evidence that colon injury in CK8^+/−^ mice was attenuated by TAK-242 treatment (Figure 9E). Furthermore, the colonic levels of TNFα and IFNγ were significantly lower in mice treated with TAK-242 than in mice without TAK-242 treatment (Figure 9F). These results suggest that TLR4/NF-kB signaling enhances the susceptibility of CK8^+/−^ mice to DSS-induced colitis.

## DISCUSSION

There is increasing evidence that CK8 may be an important signaling molecule regulating the inflammatory response. Our previous study indicated that CK8 prevented the polyubiquitination of TRAF6, thus suppressing NF-κB signaling and TLR-dependent inflammation [14]. In addition, chronic colitis was found to occur spontaneously in homozygous CK8^−/−^ FVB/N mice [9]. In the present study, we demonstrated that knockdown of CK8 increased the vulnerability of mice to DSS-induced acute colitis and promoted AOM/DSS-induced CAC at a very early stage. CK8^+/−^ mice exhibited increased colonic permeability and an abnormal gut microbiota composition, while antibiotic treatment reduced the tumor incidence in this strain. Furthermore, the TLR4-specific inhibitor TAK-242 reduced the incidence of colitis upon DSS treatment. These data indicated that CK8 prevents colitis and colonic tumorigenesis by protecting the colonic integrity and maintaining gut microbiota homeostasis.

Various lines of evidence suggest that keratins are key regulators of epithelial integrity. Keratin expression and organization are transcriptionally and post-translationally modified under different stress conditions, thus re-establishing the equilibrium in tissues. Keratins are the main sources of mechanical integrity in keratinocytes, even when just one pair of keratins is expressed [29]. Keratin 9 is required for terminal differentiation and for maintaining the structural integrity of the palmoplantar epidermis [30]. Furthermore, CK8 is needed to preserve the thymic epithelial structure and integrity (in association with CK18) [31].

Disturbances in the epithelial barrier are frequently associated with diseases of the intestines, including IBD. When epithelial cells fail to form an effective barrier, the immune system may become sensitized to luminal antigens [32] [16]. Wang *et al.* performed experiments in Caco2-BBE cells and IL-6^−/−^ mice, and found that IL-6 increased CK8 and CK18 levels in the insoluble cytoskeletal fraction and promoted the serine 431 and 73 phosphorylation of CK8, indicating that IL-6 may induce the post-translational modification of CK8 [15]. DSS treatment of IL-6^−/−^ mice enhanced their intestinal permeability, suggesting that IL-6 may regulate barrier function through CK8 [15]. In the present study, we found that CK8 was downregulated and the colonic permeability of CK8^+/−^ mice was elevated during DSS-stimulated colitis and AOM/DSS-stimulated CAC, suggesting that CK8 is a key promoter of colonic epithelial integrity.

We hypothesize that, due to the enhanced colonic permeability caused by DSS or AOM/DSS treatment, bacteria can translocate into the intestinal mucosa of CK8^+/−^ mice and induce an exacerbated inflammatory cytokine response. Various studies have indicated that bacteria are involved in the pathogenesis of CRC [33]. Imbalances in microbiota can promote colon tumorigenesis through many pathways. The enterotoxigenic *Bacteroides fragilis* can cause colitis, colonic hyperplasia, and tumor formation by activating STAT3- and TH17-dependent pathways [34]. Erdman et al. demonstrated that Rag-2^−/−^ mice developed CAC only upon infection with *Helicobacter hepaticus* [35]. In another study, vancomycin and imipenem antibiotic treatments suppressed inflammation in the CK8^−/−^ colon, indicating that bacteria in the lumen may induce colitis when there is a primary defect of the epithelium [9].

In the present study, the gut microbiota composition differed between CK8^+/−^ CRC mice and CK8^+/+^ CRC mice, and treatment with antibiotics reduced the development of CRC. Phyla *Firmicutes* and *Proteobacteria* were more abundant in CRC CK8^+/−^ mice, while phyla *Bacteroidetes* and *Verrucomicrobia* were more abundant in CRC CK8^+/+^ mice. In a study of an experimental CRC rat model, *Firmicutes*, *Proteobacteria*, and *Actinobacteria* were more abundant in the intestinal lumens of CRC rats than in those of healthy rats, whereas *Bacteroidetes* were more abundant in the healthy group [36]. Similar findings were obtained in a human study of CRC patients and cancer-free controls [37]. *Firmicutes* are the major butyrate-producing bacteria, and butyrate has been suggested to stimulate the excessive colonic epithelial cell proliferation in the APC^Min/+^MSH2^−/−^ mouse (a model of colon cancer) [38].

The abundance of *Verrucomicrobia* phylum *Akkermansia* genus was reduced in CRC CK8^+/−^ mice. A previous study suggested that healthy individuals have high levels of *Akkermansia muciniphila* (which degrades mucin), while humans with inflammatory diseases of the gastrointestinal tract and mice with obesity or type 2 diabetes have lower levels of these bacteria [39]. Interestingly, our study indicated that bacteria of the *Proteobacteria* phylum *Helicobacteraceae* family *Helicobacter* genus were significantly more abundant in CRC CK8^+/−^ mice than in CRC CK8^+/+^ mice. The abundance of *Helicobacteraceae* has been reported to be elevated in IBD patients [40], and *Helicobacter pylori* has been shown to be an independent risk factor for early and advanced colorectal neoplasms [41] and colonic adenomatous neoplasms [42]. Overall, our data indicated that the CK8 knockdown led to an imbalance of the gut microbiota composition under stress conditions, and that the gut microbiota composition was a critical contributor to the colitis and increased tumor incidence of CK8^+/−^ mice. However, to identify the commensal bacteria responsible for colorectal cancer development in CK8^+/−^ mice, a further study with a specific antibiotic will be required.

Widespread maladies (e.g., *Helicobacter pylori* infection and gastric cancer) may chronically induce infection and inflammation and subsequently activate TLR4 [43]. Our study demonstrated that, upon DSS treatment, the serum concentration of LPS (the ligand for TLR4) was much higher in CK8^+/−^ mice than in CK8^+/+^ mice. NF-kB activity was also enhanced in CK8^+/−^ mice following DSS or AOM/DSS treatment. There is sufficient evidence that TLR4 is a key inducer of colitis and CAC. TLR4 is overexpressed in colon tumors resulting from chronic ulcerative colitis in humans and from inflammation in animals [44]. In acute colitis, TLR4 strongly stimulates the expression of COX2 [45]. Inflammation-induced colon cancer was prevented by the genetic ablation of *Tlr4* in mice [44]. Our study suggested that CK8 negatively regulates TLR/NF-kB signaling, and that CK8^+/−^ mice are more susceptible to LPS/TLR4-induced inflammatory responses than WT mice. The specific TLR4 inhibitor, TAK-242, significantly attenuated the severity of DSS-induced colitis in CK8^+/−^ mice, suggesting that TLR4/NF-kB signaling may be involved in the inflammatory response to microbiota and may further promote tumor formation.

In summary, our results support the concept that, under stress conditions, intact CK8 expression is critical for maintaining the intestinal epithelial barrier and gut microbiota homeostasis. Dysregulation of CK8 leads to uncontrolled inflammatory responses and increased CAC development.

## MATERIALS AND METHODS

### Ethics statement

Investigation has been conducted in accordance with the ethical standards and according to the Declaration of Helsinki and according to national and international guidelines and has been approved by the Animal Ethics Committee of the Beijing Institute of Radiation Medicine.

### Cell lines and reagents

HT29 cells were maintained in DMEM (Gibco Invitrogen, CA) with 10% fetal calf serum (FCS). All the cells were cultured in a 37°C incubator with 5% CO_2_ in the presence of 2 mM glutamine, 100 IU/ml penicillin, 100 mg/ml streptomycin, 2 g/l sodium bicarbonate and 10 mM HEPES.

### Mice

All mice were bred in a specific pathogen-free facility, and all animal experiments were approved by the Animal Ethics Committee of the Academy of Military Medical Science.

### DSS-induced colitis

Colitis was induced in CK8^+/+^ and CK8^+/−^ mice by administering 5% (wt/vol) DSS (36-50 kD; MP Biomedical) that was dissolved in drinking water for five consecutive days as described previously, followed by regular water [45].

### Western blotting analysis

For Western blotting, cells were lysed with M-PER^®^ Mammalian Protein Extraction Reagent (Pierce, Rockford, IL, USA). Then, Western blot analysis was performed according to standard procedures. Antibodies were used at the following concentrations: CK8 (ab53280, Abcam), 1:3000; GAPDH (sc-47778, Santa Cruz), 1:1000. Chemiluminescent detection was conducted using supersignal substrate (Pierce) according to the manufacturer’s specifications.

### Quantitative real-time RT-PCR

Total RNA was reverse-transcribed and amplified using reverse transcription and PCR kits, respectively (Promega Corp., Madison, WI, U.S.). Real-time RT-PCR was performed by Bio-Rad IQ5 (Bio-Rad, U.S.). The abundance of mRNA of each gene was normalized to GAPDH. The sequences of the primers are provided in Supplementary Table 1.

### Intestinal permeability

Mice were anaesthetised and 100 microliters of 80 mg/ml FITC-dextran solution was delivered via rectal enema. Mice were inverted for 30 min prior to sacrificing and harvesting blood via cardiac puncture. Blood was allowed to clot followed by centrifugation and serum harvesting. Samples were read at 480 and 520 nm on a Microplate Reader (EnSpire, PerkinElmer, USA).

### Collection of tissue specimens

Human tissue specimens were obtained from surgical resections and biopsies in accordance with human studies guidelines and approval by the ethics committee of the Beijing Institute of Radiation Medicine. Clinical data was recorded from medical records and then the identifying information was removed. Tissues for immunohistochemical use were fixed in buffered formalin and embedded in paraffin using standard methods. Normal colon specimens were obtained from patients undergoing colonoscopy and who had normal colonoscopic exams and normal histology.

### Immunohistochemistry

The slides were baked at 60°C 2 hours and then deparaffinized by washing in xylene (2 × 10 min). Sections were hydrated through graded alcohol and endogenous peroxidase activity was quenched by incubating the sections in 3% H_2_O_2_ in methanol for 30 min. After further washing with tap water for 15 min, antigen retrieval was done by heating the slides in citrate buffer (0.01 M, pH 6.0) at 92∼98°C for 15 min. After heating, the samples were allowed to cool at room temperature. This was followed by washing with phosphate-buffered saline (PBS) (3 × 5 min). Non-specific binding was blocked by incubating the sections with 10% normal goat serum for 30 min. Sections were then incubated with anti-CK8 antibody (1:300) at 4°C overnight. Then slides were washed with phosphate buffered saline (3 × 5 min) and incubated with secondary antibody auxiliary regent for 30 min. Then sections were washed with phosphate buffered saline (3 × 5 min) and incubated with secondary antibody (PV-9001, ZSGB-Bio, China) 30 min.Then sections washed with PBS (3 × 5 min) followed by treatment with DAB reagent (Wuhan Boster Biological Technology Ltd., China). After washing with PBS, counterstaining was done using haematoxylin. With further washing in tap water, sections were dehydrated in graded alcohol and covered with cover glass.

### AOM/DSS-induced colon tumorigenesis

8–12-week-old mice were injected intraperitoneally (i.p.) with 10 mg/kg azoxymethane (Sigma). Water containing 2% dextran sulfate sodium (MP Biomedicals, 36-50 kD) was fed on day 5 for 5 days followed by 16 days of water. This was repeated twice. Mice were sacrificed 3 weeks after the end of the third cycle of DSS or at the end of 10 weeks. After sacrifice, colons were harvested, flushed of feces and longitudinally slit open to grossly count tumors with the aid of a magnifier and stereomicroscope.

### Depletion of gut microbiota

Mice were initially given a four week treatment of 1 g/L ampicillin, 0.5 g/L vancomycin, 1 g/L metronidazole and 1g/L neomycin for 4 weeks. Due to only modest intestinal depletion as assayed by fecal cultures, mice were subsequently switched over to a different antibiotic cocktail of 2 g/L streptomycin, 0.17 g/L gentamycin, 0.125 mg/L ciprofloxacin and 1g/L bacitracin which was maintained for the duration of the experiment. Intestinal depletion was assessed by collecting feces, homogenizing in PBS, serially diluting and plating on trypticase soy agar with 10% sheep blood (Fisher Scientific) for 48 hours at 37°C aerobically and in anaerobic chamber (AnaeroPack System).

### Metagenomic analysis of fecal sample microbiota

Genomic DNA was isolated from approximately 100 mg of fecal samples which were harvested from CK8^+/−^ and littermate WT mice at the end of the third DSS cycle using the TIANamp Stool DNA kit (DP328-02, TIANGEN, China) following the manufacturer’s instruction. The 16S ribosomal RNA gene in the fecal samples was sequenced by Anoroad Ltd.(China). DNA library construction was performed using the IlluminaHiseqTM2500. Adaptor contamination, low-quality reads, and host contaminating reads were removed from the raw sequencing reads sets. 0.366 million (CK8^+/−^ mice) and 0.357 million (WT- mice) high-quality reads per sample were generated for further analyses. The reads with sequence similarity greater than 97% were classified as a class of OTUs (Operational Taxonomic Units). Then Cluster analysis of OTUs was carried out by Uclust 1.1.579.

### Examination of NF-κB activity

Extraction of nuclear protein from different colon tissues which treated by DSS or AOM/DSS. Then exam the NF-κB activity using the NF-κB p65 Transcription Factor Assay Kit(ab133112 abcam) following the manufacturer’s instruction. Read absorbance at 450 nm by Microplate Reader (EnSpire, PerkinElmer, USA).

### Inflammation score

Colons from mice were fixed in 10%(vol/vol) neutral formalin. Paraffin-embedded tissues were cut into sections and stained with hematoxylin and eosin. Inflammation score of histopathologic changes in the colonic were semi-quantified according to a modified scoring system [46]:(a) cellular infiltration in the lamina propria (scored from 0 to 4); (b) thickness of mucosa (scored from 0 to 4); (c) mucosal damage(scored from 0 to 4); (d) submucosal oedema (scored from 0 to 4); (e) lesion range(scored from 0 to 4).

### Statistical analysis

All experiments were performed at least three times. Data were reported as means ± SD and the statistical significance was assessed by one-way analysis of variance followed by Students–Newman–Keuls tests. A value of *p* ≤ 0.05 was considered to be significant. Kaplan-Meier curves were constructed to compare survival. Diferences in survival were evaluated with the Mantel-Cox test. A value of *p* ≤ 0.05 was considered to be significant.

## SUPPLEMENTARY MATERIALS FIGURES AND TABLES





## Abbreviations

CK8: keratin 8
CRC: colorectal cancer
CAC: colitis-associated colorectal cancer
AOM: azoxymethane
DSS: dextran sodium sulfate
TNFα: tumor necrosis factor alpha
IBD: Inflammatory bowel disease
WT: wild type
TLRs: Toll-like receptor
IF: Intermediate filaments
