# Supplementary material for: Keratin 8 reduces colonic permeability and maintains gut microbiota homeostasis, protecting against colitis and colitis-associated tumorigenesis
Source: Oncotarget. 2017 May 27;8(57):96774–90. doi: 10.18632/oncotarget.18241 (PMC5722522; doi:10.18632/oncotarget.18241)
Supplement: Supplementary file 1 [file oncotarget-08-96774-s001.pdf]

Keratin 8 reduces colonic permeability and maintains gut microbiota homeostasis, protecting against colitis and colitis-associated tumorigenesis

Supplementary Materials

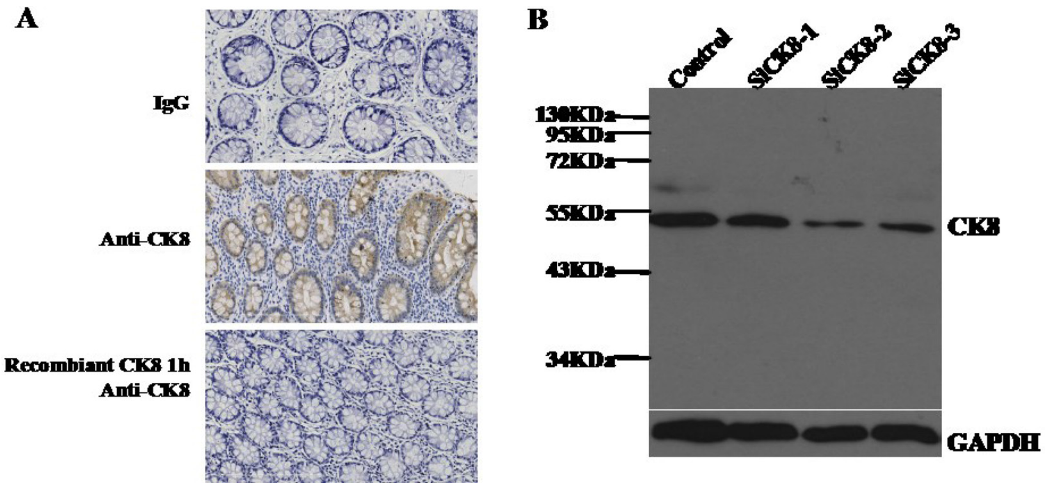

Supplementary Figure 1: The validation of the specificityof CK8 antibody. (**A**) Immunohistochemical staining for colon cancer specimens incubated with IgG or CK8-specific antibody. To validate the specificity, monoclonal antibody to CK8 was pre-incubated with recombinant CK8 protein for 1 h prior to applying to tissue. (**B**) HT29 cells were transfected with control or CK8 siRNA, and the expression of CK8 was investigated with Western blot analysis.

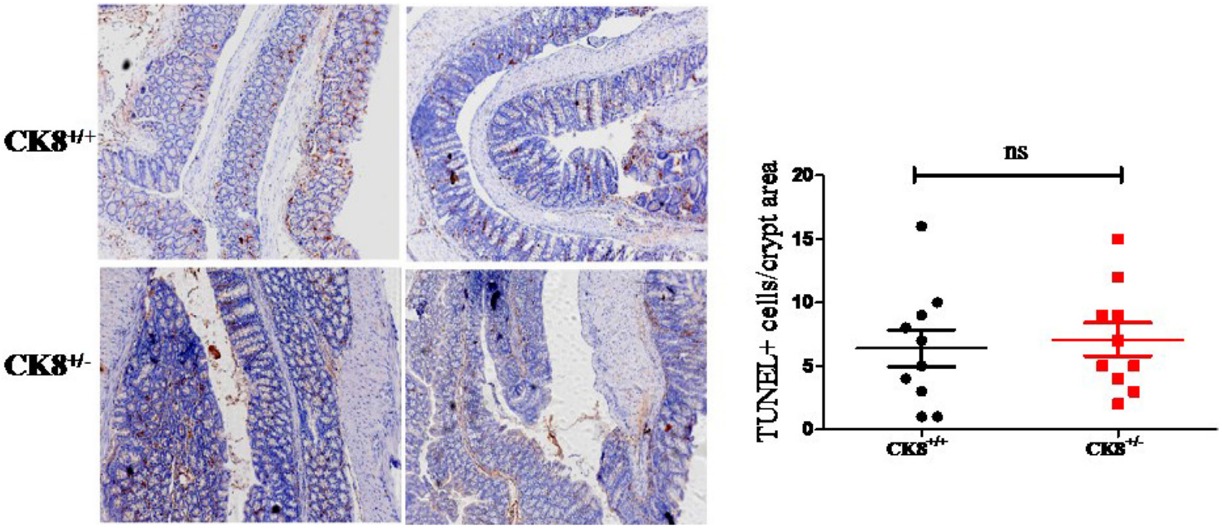

Supplementary Figure 2: The apoptosis analysis in CK8$^{+/-}$ and CK8$^{+/+}$ colons with tumors. CK8$^{+/+}$ mice and CK8$^{+/-}$ mice were treated with AOM/DSS as Material and Methods described. The apoptosis in colons were measured using TUNEL assay.

Supplementary Table 1: Real-time quantitative PCR primers

β-catenin	Forward primer:ATGGAGCCGGACAGAAAAGC
	Reverse primer:CTTGCCACTCAGGGAAGGA
E-cadherin	Forward primer:CAGGTCTCCTCATGGCTTTGC
	Reverse primer:CTTCCGAAAAGAAGGCTGTCC
MMP2	Forward primer:CAAGTTCCCCGGCGATGTC
	Reverse primer:TTCTGGTCAAGGTCACCTGTC
MMP9	Forward primer:CTGGACAGCCAGACACTAAAG
	Reverse primer:CTCGCGGCAAGTCTTCAGAG
TNFα	Forward primer:CCTCTCTCTAATCAGCCCTCTG
	Reverse primer:GAGGACCTGGGAGTAGATGAG
IL-1α	Forward primer:GTGTTGCTGAAGGAGTTGCC
	Reverse primer:CGACTTTGTTCTTTGGTGGC
IL-1β	Forward primer:GCTTCAGGCAGGCAGTA
	Reverse primer:TGAGTCACAGAGGATGGG
IFNγ	Forward primer:TCGGTAACTGACTTGAATGTCCA
	Reverse primer:TCGCTTCCCTGTTTTAGCTGC
IL-4	Forward primer:GGTCTCAACCCCCAGCTAGT
	Reverse primer:GCCGATGATCTCTCTCAAGTGAT
IL-17	Forward primer:TTTAACTCCCTTGGCGCAAAA
	Reverse primer:CTTTCCCTCCGCATTGACAC
CCL4	Forward primer:TTCCTGCTGTTTCTCTTACACCT
	Reverse primer:CTGTCTGCCTCTTTTGGTCAG
iNOS	Forward primer:ACATCGACCCGTCCACAGTAT
	Reverse primer:CAGAGGGGTAGGCTTGTCTC
KC	Forward primer:CTGGGATTCACCTCAAGAACATC
	Reverse primer:CAGGGTCAAGGCAAGCCTC

Supplementary Table 2: Relative contribution in the gut microbiota of CK8$^{+/-}$ and CK8$^{+/+}$ CRC mice. See Supplementary_Table_2
