# Supplementary material for: Keratin 8 reduces colonic permeability and maintains gut microbiota homeostasis, protecting against colitis and colitis-associated tumorigenesis
Source: Oncotarget. 2017 May 27;8(57):96774–90. doi: 10.18632/oncotarget.18241 (PMC5722522; doi:10.18632/oncotarget.18241)
Supplement: Supplementary file 2 [file oncotarget-08-96774-s002.doc]

Relative contribution of the predominant phyla in the gut microbiota of CK8+/- and CK8+/+ CRC mice.

| **name** | **CK8^+/--^1** | **CK8^+/--^2** | **CK8^+/-^-3** | **CK8^+/--^4** |  | **CK8^+/+^-1** | **CK8^+/+^-2** | **CK8^+/+^-3** | **CK8^+/+^-4** |
| --- | --- | --- | --- | --- | --- | --- | --- | --- | --- |
| Bacteroidetes | 0.509846172 | 0.617180537 | 0.580185317 | 0.470357931 |  | 0.691574807 | 0.75524675 | 0.644978673 | 0.607466471 |
| Firmicutes | 0.439403045 | 0.324107562 | 0.363660347 | 0.468938756 |  | 0.247342387 | 0.160726463 | 0.257244989 | 0.322099869 |
| Verrucomicrobia | 0.000220722 | 0.000211975 | 0.000140446 | 0.00014573 |  | 0.051005022 | 0.073553663 | 0.058568951 | 0.060817967 |
| Proteobacteria | 0.029799667 | 0.05319804 | 0.022461734 | 0.028351158 |  | 0.006503254 | 0.007990002 | 0.009127718 | 0.005176035 |
| Candidate division TM7 | 0.010839421 | 0.003005496 | 0.014749291 | 0.024874226 |  | 0.000294521 | 6.24856E-05 | 0.016037326 | 0.001513763 |
| Cyanobacteria | 0.000699318 | 0.000749481 | 0.004087474 | 0.00524412 |  | 0.001268706 | 0.001510521 | 0.007510771 | 0.002132462 |
| Actinobacteria | 0.002869387 | 0.000259921 | 0.010579487 | 0.001107116 |  | 0.000480799 | 0.000263526 | 0.003219517 | 0.00055986 |
| Tenericutes | 0.005345408 | 0.000855469 | 0.003339234 | 0.000715602 |  | 0.001522951 | 0.00047815 | 0.00324319 | 8.91498E-06 |
| Deferribacteres | 0.000976859 | 0.00043152 | 0.00079667 | 0.00026536 |  | 7.55182E-06 | 0.000168439 | 6.88667E-05 | 0.000224657 |

Relative contribution of the predominant classes in the gut microbiota of CK8+/- and CK8+/+ CRC mice.

| **name** | **CK8^+/--^1** | **CK8^+/--^2** | **CK8^+/-^-3** | **CK8^+/--^4** |  | **CK8^+/+^-1** | **CK8^+/+^-2** | **CK8^+/+^-3** | **CK8^+/+^-4** |
| --- | --- | --- | --- | --- | --- | --- | --- | --- | --- |
| Bacteroidia | 0.51317309 | 0.622627941 | 0.596369331 | 0.485408954 |  | 0.693360886 | 0.759330127 | 0.672942025 | 0.604213259 |
| Clostridia | 0.434328125 | 0.314539269 | 0.361871892 | 0.48029092 |  | 0.227268712 | 0.144600231 | 0.246352128 | 0.318629964 |
| Verrucomicrobiae | 0.000219973 | 0.000213596 | 0.00014158 | 0.000148062 |  | 0.051140193 | 0.073952674 | 0.061149883 | 0.060494219 |
| Epsilonproteobacteria | 0.025921632 | 0.048132816 | 0.010126727 | 0.024640198 |  | 0.004578312 | 0.002922707 | 0.002780472 | 0.00012947 |
| Bacilli | 0.007184322 | 8.75E-03 | 9.54E-03 | 0.003129494 |  | 0.019691826 | 0.01369848 | 0.001635757 | 0.012922128 |
| Actinobacteria | 0.002888247 | 0.000261909 | 1.09E-02 | 0.001141873 |  | 4.82E-04 | 0.000264956 | 0.00336139 | 0.000556897 |
| Mollicutes | 0.005380542 | 0.000862012 | 0.003425253 | 0.000738067 |  | 1.53E-03 | 0.000480744 | 0.003386106 | 8.86778E-06 |
| Alphaproteobacteria | 0.000750108 | 0.000376336 | 0.002533545 | 0.001698227 |  | 0.001188779 | 0.002286267 | 0.004350034 | 0.001723896 |
| Deltaproteobacteria | 6.92E-03 | 0.003318365 | 0.003743188 | 0.002337586 |  | 1.87E-04 | 0.000210326 | 0.002736747 | 0.000631386 |
| Deferribacteres | 9.83E-04 | 0.00043482 | 8.17E-04 | 0.00027369 |  | 7.57184E-06 | 0.000169353 | 7.19014E-05 | 0.000223468 |
| Betaproteobacteria | 2.25E-03 | 4.83E-04 | 5.84E-04 | 0.000192929 |  | 5.68E-04 | 0.002084136 | 0.001233558 | 0.000466445 |

Relative contribution of the predominant orders in the gut microbiota of CK8+/- and CK8+/+ CRC mice.

| **name** | **CK8^+/--^1** | **CK8^+/--^2** | **CK8^+/-^-3** | **CK8^+/--^4** |  | **CK8^+/+^-1** | **CK8^+/+^-2** | **CK8^+/+^-3** | **CK8^+/+^-4** |
| --- | --- | --- | --- | --- | --- | --- | --- | --- | --- |
| Bacteroidales | 0.521021635 | 0.629609271 | 0.479994523 | 0.486925061 |  | 0.689974785 | 0.758983908 | 0.61088398 | 0.669212373 |
| Clostridiales | 0.44096857 | 0.316611939 | 0.465344705 | 0.48175851 |  | 0.226142579 | 0.14452884 | 0.322147747 | 0.244966173 |
| Verrucomicrobiales | 0.000127302 | 0.000256737 | 0.000182579 | 0.000148515 |  | 0.050886789 | 0.073918955 | 0.061162096 | 0.060805859 |
| Campylobacterales | 0.009105467 | 0.030253939 | 0.041143381 | 0.024685002 |  | 0.004506075 | 0.002921374 | 0.000130899 | 0.00270857 |
| Lactobacillales | 0.007066396 | 0.008798392 | 0.008335616 | 0.002414497 |  | 0.01957416 | 0.013659472 | 0.001289265 | 0.016097969 |
| Bifidobacteriales | 0.009442706 | 0.003098821 | 1.73885E-05 | 0.000702072 |  | 2.00915E-05 | 1.0921E-05 | 0.000104002 | 9.60739E-05 |
| Anaeroplasmatales | 0.001987703 | 0.00622845 | 0.000717276 | 0.000182269 |  | 2.51144E-06 | 0 | 3.58627E-06 | 8.93711E-06 |
| Coriobacteriales | 0.000241204 | 0.000172014 | 0.000119546 | 0.000384789 |  | 0.000424433 | 0.000163815 | 0.000376559 | 0.003127988 |
| Desulfovibrionales | 0.007026195 | 0.003347856 | 0.003271213 | 0.002333489 |  | 0.007900989 | 0.003560254 | 0.002698671 | 0.002316946 |
| Deferribacterales | 0.00073478 | 0.001147616 | 0.000371679 | 0.000274528 |  | 7.53432E-06 | 0.000169276 | 0.000225935 | 7.14969E-05 |
| Burkholderiales | 0.002278042 | 0.000474964 | 0.000502093 | 0.00019127 |  | 0.000560051 | 0.002083185 | 0.000977259 | 0.000587615 |

Relative contribution of the predominant families in the gut microbiota of CK8+/- and CK8+/+ CRC mice.

| **name** | **CK8+/--1** | **CK8+/--2** | **CK8+/--3** | **CK8+/--4** |  | **CK8+/+-1** | **CK8+/+-2** | **CK8+/+-3** | **CK8+/+-4** |
| --- | --- | --- | --- | --- | --- | --- | --- | --- | --- |
| Bacteroidaceae | 0.359470724 | 0.432784718 | 0.331979733 | 0.170796848 |  | 0.521734828 | 0.359760268 | 0.249856177 | 0.231065378 |
| Prevotellaceae | 0.067128653 | 0.04257281 | 0.143640539 | 0.057823882 |  | 0.151415094 | 0.293096114 | 0.258169427 | 0.149567044 |
| Rikenellaceae | 0.116983027 | 0.053804934 | 0.09474322 | 0.176777936 |  | 0.040624459 | 0.024420189 | 0.083456425 | 0.148044286 |
| Lachnospiraceae | 0.165692478 | 0.168478374 | 0.110298863 | 0.290213461 |  | 0.099350965 | 0.077223799 | 0.179678794 | 0.16406089 |
| Ruminococcaceae | 0.189479763 | 0.220038971 | 0.1946598 | 0.259539774 |  | 0.064243714 | 0.054720608 | 0.097402757 | 0.087425433 |
| Verrucomicrobiaceae | 0.000286231 | 0.000347959 | 0.000368804 | 0.000265469 |  | 0.083549816 | 0.145788796 | 0.110488092 | 0.136771853 |
| Helicobacteraceae | 0.018454354 | 0.037565677 | 0.081606758 | 0.023618658 |  | 0.002609004 | 0.005540987 | 0.000233227 | 0.008173544 |
| Porphyromonadaceae | 0.022933615 | 0.013525175 | 0.016824506 | 0.009741892 |  | 0.003311149 | 0.012686652 | 0.013196766 | 0.023439424 |
| Lactobacillaceae | 0.015878277 | 0.011914124 | 0.016793772 | 0.004291742 |  | 0.032117733 | 0.025674853 | 0.002303117 | 0.035907951 |
| Bifidobacteriaceae | 0.021231295 | 0.004199868 | 3.51242E-05 | 0.001254942 |  | 3.29878E-05 | 2.15393E-05 | 0.000187877 | 0.000216101 |
| Anaeroplasmataceae | 0.004469218 | 0.008441491 | 0.001448874 | 0.000325802 |  | 4.12347E-06 | 0 | 6.47853E-06 | 0.00634734 |
| Coriobacteriaceae | 0.000542332 | 0.000233133 | 0.000241479 | 0.000687805 |  | 0.000696867 | 0.00032309 | 0.000680246 | 0.007035848 |
| Desulfovibrionaceae | 0.015797931 | 0.004537388 | 0.006607746 | 0.004171074 |  | 0.00029689 | 0.000409247 | 0.003932468 | 0.00178409 |
| Deferribacteraceae | 0.001652104 | 0.001555378 | 0.00075078 | 0.000490715 |  | 1.23704E-05 | 0.000333859 | 0.000408147 | 0.000160819 |

Relative contribution of the predominant genera in the gut microbiota of CK8+/- and CK8+/+ CRC mice.

| **name** | **CK8+/--1** | **CK8+/--2** | **CK8+/--3** | **CK8+/--4** |  | **CK8+/+-1** | **CK8+/+-2** | **CK8+/+-3** | **CK8+/+-4** |
| --- | --- | --- | --- | --- | --- | --- | --- | --- | --- |
| Bacteroides | 0.451568613 | 0.560786646 | 0.408087285 | 0.271712482 |  | 0.601719084 | 0.396207825 | 0.266228956 | 0.307392354 |
| Rikenella | 0.147324619 | 0.067607272 | 0.107262153 | 0.266052436 |  | 0.045010142 | 0.025978118 | 0.104289537 | 0.175123207 |
| Prevotella | 0.084539642 | 0.053493822 | 0.162620539 | 0.087025479 |  | 0.145602237 | 0.311794696 | 0.322615901 | 0.176924495 |
| Parabacteroides | 0.0288692 | 0.016985983 | 0.019037678 | 0.014661638 |  | 0.003664041 | 0.013496019 | 0.01648296 | 0.027726751 |
| Oscillospira | 0.147879932 | 0.176518682 | 0.155000497 | 0.179680737 |  | 0.034100254 | 0.026298906 | 0.076472517 | 0.046096318 |
| Blautia | 0.000980225 | 0.002750111 | 0.001461378 | 0.00472175 |  | 0.002307158 | 0.00209658 | 0.003736192 | 0.001640777 |
| Akkermansia | 0.00036047 | 0.00043722 | 0.000417537 | 0.000399533 |  | 0.09256958 | 0.155089649 | 0.138069081 | 0.161788922 |
| Helicobacter | 0.023240813 | 0.047202232 | 0.0923899 | 0.0355463 |  | 0.005049798 | 0.005894484 | 0.000291447 | 0.003326794 |
| Odoribacter | 0.005824432 | 0.001460314 | 0.002405806 | 0.009171091 |  | 0.003810237 | 0.007068798 | 0.01098999 | 0.014083335 |
| Lactobacillus | 0.019996585 | 0.0149704 | 0.019012824 | 0.006459111 |  | 0.035585059 | 0.027312826 | 0.002878041 | 0.042475909 |
| Anaeroplasma | 0.005628387 | 0.010606948 | 0.001640322 | 0.000490336 |  | 4.56863E-06 | 0 | 8.09576E-06 | 2.37794E-05 |
| Ruminococcus | 0.057630892 | 0.039332278 | 0.017730391 | 0.109532486 |  | 0.020152227 | 0.015455118 | 0.028804702 | 0.025580663 |
| Butyricimonas | 6.32403E-06 | 2.1861E-05 | 9.94135E-06 | 6.05353E-06 |  | 0.00159902 | 0.006392851 | 0.008443874 | 0.000101062 |
| Dorea | 0.006254466 | 0.002124887 | 0.005442887 | 0.008263062 |  | 0.008497652 | 0.006478776 | 0.015774582 | 0.015605215 |
| Desulfovibrio | 0.019895401 | 0.005701344 | 0.007480863 | 0.006277506 |  | 0.000328941 | 0.000435355 | 0.004914124 | 0.00211042 |
